# Object Identification and Safe Route Recommendation Based on Human Flow for the Visually Impaired

**DOI:** 10.3390/s19245343

**Published:** 2019-12-04

**Authors:** Yusuke Kajiwara, Haruhiko Kimura

**Affiliations:** Department of Production Systems Engineering and Sciences, Komatsu University, Awazu Campus, Komatsu ⍑923-0921, Japan; haruhiko.kimura@komatsu-u.ac.jp

**Keywords:** human flow, visually impaired, object identification, safe route recommendation

## Abstract

It is difficult for visually impaired people to move indoors and outdoors. In 2018, world health organization (WHO) reported that there were about 253 million people around the world who were moderately visually impaired in distance vision. A navigation system that combines positioning and obstacle detection has been actively researched and developed. However, when these obstacle detection methods are used in high-traffic passages, since many pedestrians cause an occlusion problem that obstructs the shape and color of obstacles, these obstacle detection methods significantly decrease in accuracy. To solve this problem, we developed an application “Follow me!”. The application recommends a safe route by machine learning the gait and walking route of many pedestrians obtained from the monocular camera images of a smartphone. As a result of the experiment, pedestrians walking in the same direction as visually impaired people, oncoming pedestrians, and steps were identified with an average accuracy of 0.92 based on the gait and walking route of pedestrians acquired from monocular camera images. Furthermore, the results of the recommended safe route based on the identification results showed that the visually impaired people were guided to a safe route with 100% accuracy. In addition, visually impaired people avoided obstacles that had to be detoured during construction and signage by walking along the recommended route.

## 1. Introduction

It is difficult for visually impaired people to move indoors and outdoors. In 2018, world health organization (WHO) reported that there were about 253 million people around the world who were moderately visually impaired in distance vision [1]. Visually impaired people generally use braille blocks, guide dogs, and white canes to move indoors and outdoors. In addition to these auxiliary devices, a navigation system that combines positioning and obstacle detection has been actively researched and developed [2]. The radiofrequency identifier (RFID) can be positioned with an error of about 10 mm [3,4,5]. The user charges for installing RFID tags on the floor. However, RFID is the most accurate of current positioning methods. Obstacles are mainly detected by applying simultaneous localization and mapping (SLAM) [6] to the camera images. These obstacle detection methods can be detected with high accuracy in low-traffic passages. However, when these obstacle detection methods are used in high-traffic passages, since many pedestrians cause an occlusion problem that obstructs the shape and color of obstacles, these obstacle detection methods significantly decrease in accuracy.

To solve this problem, in this research we develop an application “Follow me!”. The application recommends a safe route by machine learning the gait and walking route of many pedestrians obtained from the monocular camera images of a smartphone. There are generally three obstacles in the passage:

- Obstacles that must be detoured, such as areas under construction or signage;
- Obstacles that require pedestrians to go up and down, such as steps;
- Obstacles that change position dynamically, such as oncoming pedestrians.

This research shows that hidden objects can be detected using human flow. This fact is ground-breaking. Pedestrians generally walk to a safe passage without obstacles. The gait of the pedestrian changes when pedestrians go up and down steps. Therefore, the gait and walking route of many pedestrians are acquired using image processing, and a safe route is recommended by the machine learning of these data.

The proposed method is used in combination with existing obstacle detection methods. In high-traffic passages, visually impaired people are navigated to their destination using the proposed methods. In low-traffic passages, visually impaired people are navigated to their destination using existing methods. By switching automatically between the proposed method and the existing method according to the situation, visually impaired people can walk comfortably in both high-traffic passages and low-traffic passages.

The proposed method is novel in that it recommends a safe route from the pedestrian’s gait and the walking route obtained using a monocular camera. The effectiveness of the proposed method is as follows:

- The proposed method is robust against occlusion problems caused by pedestrians;
- The system design is simple and the amount of calculation is small since the proposed method only needs to detect pedestrians ahead;
- The proposed method is implemented on a smartphone. A pedestrian’s gait and walking route are detected by a monocular camera. Therefore, special sensors such as RGB-D sensors that obtain the color and depth are not required. Therefore, the proposed method can be expected to spread to visually impaired people.

As a result of the experiment, pedestrians walking in the same direction as visually impaired people, oncoming pedestrians, and steps are identified with an average accuracy of 0.92 based on the gait and walking route of pedestrians acquired from monocular camera images. Furthermore, the recommended results of a safe route based on the identification results show that the visually impaired people are guided to a safe route with 100% accuracy. In addition, visually impaired people avoid obstacles that have to be detoured during construction and signage by walking along the recommended route. 

Section 2 introduces related research on positioning, object identification, and navigation. Section 3 describes the configuration of the proposed method. Section 4 explains the evaluation experiment of the proposed method and its results. Section 5 presents conclusions and future plans.

## 2. Related Research

### 2.1. Positioning

By using related research on positioning, visually impaired people can grasp their own position even in a crowd. Moreover, RGB-D sensors are often used in related research to implement SLAM. However, the RGB-D sensor is a special sensor and is not installed in general smartphones. RGB-D sensors are difficult to use outdoors. 

Most of the positioning methods improve their accuracy by measuring the position roughly by radio, such as with the global positioning system (GPS) [7], Bluetooth Low Energy (BLE) [8], Ultra Wide Band (UWB) [9], or RFID [3,4,5], and then correcting the position by dead reckoning or SLAM. SLAM is often used for obstacle detection. GPS measures the outdoor position from the radio waves of multiple satellites based on the principle of triangulation. The accuracy of GPS changes depending on the weather. In addition, indoor positioning is difficult because buildings absorb and reflect radio waves. BLE measures the indoor position by the received signal strength indicator (RSSI) obtained from each beacon. Each beacon is mainly installed indoors. However, when BLE is used in high-traffic passages, the signal is absorbed and reflected by people, so BLE significantly decreases in accuracy. RFID measures the position with an error of about 10 cm [3,4,5]. The user charges for installing RFID tags on the floor. However, RFID is more accurate than other wireless positioning methods. RFID can be positioned both indoors and outdoors. The accuracy of positioning is improved by correcting the position using dead reckoning or SLAM. Dead reckoning [10] corrects the position based on walking patterns such as walking time, walking direction, and walking speed. The walking pattern is obtained from acceleration, angular velocity, and geomagnetism. SLAM extracts key points in an image and tracks the key points to calculate the camera’s internal and external parameters. Internal parameters include camera focal length and lens distortion. External parameters include camera posture parameters. In related research, a navigation system using BLE beacons and SLAM with Google Tango was developed [8]. Google Tango has an RGB-D camera and an inertial sensor. The pose (position and direction) of the device that moves with the pedestrian can be estimated from the data obtained from these sensors. An indoor localization system (ANSVIP) was developed using ARCore-compatible smartphones [11]. The PERCEPT-V was developed by integrating a background extraction module based on robust principle component analysis (RPCA) into the localization algorithm. PERCEPT-V extracts high-quality key points in the background so that the position can be measured in the crowd [12]. 

### 2.2. Obstacle Detection and Identification

Most related research detects obstacles in low-traffic passages. However, the accuracy of these methods is significantly decreased in high-traffic passages, since many pedestrians cause an occlusion problem that obstructs the shape and color of obstacles. Therefore, visually impaired people are demanding a method for detecting obstacles in high-traffic passages. A small device that can be carried by visually impaired people cannot process a large amount of computations such as deep learning in real time. Processing with a large amount of computations is performed in real time by uploading data such as images acquired by the device to the server and downloading the results processed on the server to the device. 

Obstacles are detected by sensors such as ultrasonic sensors [13,14,15], RGB-D sensors [16,17,18], and stereo cameras [19,20] attached to shoes, heads, and white canes. UltraCane/BatCane is a system that detects obstacles using only a single ultrasonic sensor attached to a white cane [15]. However, obstacle detection using an ultrasonic sensor cannot identify the type of obstacle because it is difficult to obtain the shape and color of the obstacle. Image processing can identify the type of object from the shape and color of the obstacle. Therefore, visually impaired people can avoid obstacles according to the type of obstacle. We proposed a method that can detect a staircase with an accuracy of about 95% by using a stereo camera and acquiring the Hough transform and phase-only correlation methods [19]. In addition, a stereo camera was attached to a helmet to estimate the background of geometric scenes, such as street poles and bicycles, and to detect and track common static and dynamic objects in the foreground of the scene [20]. There is research to detect human faces using Android [21]. In related research, RGB-D sensors were used to detect puddles and stereo sound feedback was used to guide the visually impaired people to the safe route [17]. Co-Robotic Cane [16] was proposed, which uses RGB-D cameras to estimate device posture and identify objects in indoor environments. Co-Robotic Cane identifies indoor structures such as stairs and doorways, and objects such as tables and chairs by Gaussian mixture model (GMM)-based pattern identification. A system based on the deep convolutional neural network (DCNN) identifies 11 outdoor objects, such as cars, people, stairs, and trees [18]. First, a visually impaired person uploads captured images to the server. The server identifies 11 objects using DCNN. The identification result is downloaded to the device of the visually impaired person. Based on the identification result, the device guides the visually impaired person to a safe passage.

### 2.3. Navigation

Visually impaired people are mainly navigated by voice or vibration. Voice conveys detailed information. Vibration conveys simple information instantly. These methods are used according to the situation.

There are many systems for visually impaired people to navigate through tactile and auditory senses. Visually impaired people acquire information on the surrounding environment by making full use of hearing, touch, smell, and taste other than vision. Then, mapping is performed based on the information, and visually impaired people go to the destination. Mapping [22] is a very important procedure for visually impaired people in that it creates a conceptual model of the surrounding space and surrounding objects. Visually impaired people understand the surrounding environment by mapping [23]. But mapping is a time-consuming task. Therefore, related research supports mapping using devices that convert visual information into the five different senses. The electro-tactile display uses a mechanical element or an electro-tactile interface to convey camera images tactilely [24]. Virtual acoustic space uses reverberation to recognize characteristics of 3D space for visually impaired people [25]. Tactile sensation is faster than hearing [26]. Simple information is transmitted to the visually impaired person by tactile sense, and information that is complicated and cannot be transmitted by tactile sense is transmitted to the visually impaired person by sound. 

## 3. Object Identification and Safe Route Recommendation Based on Human Flow 

### 3.1. Outline

**Figure 1:**
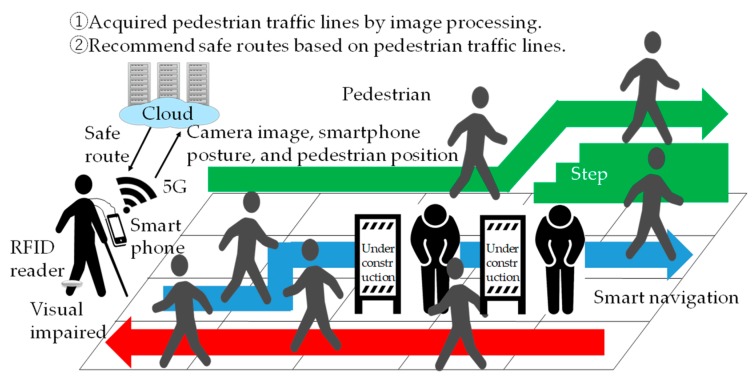
Overview of the proposed system “Follow me!”.

We propose a “Follow me!” system that recommends a safe route based on the gait and the walking route of pedestrians. Figure 1 shows the proposed system. 

**Figure 2:**
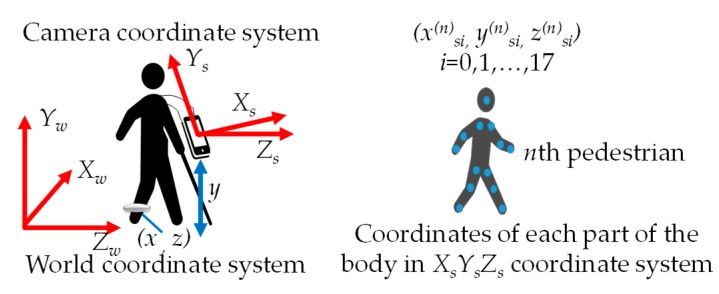
Coordinate system of visually impaired people, smartphones, and pedestrians.

Figure 2 shows the relationship between the world coordinate system *X_w_Y_w_Z_w_* and the camera coordinate system *X_s_Y_s_Z_s_*, the coordinates of the visually impaired people (*x*, *z*), and the coordinates of the pedestrian’s skeleton *i*. 

In the proposed method, the position (*x*, *z*) of the visually impaired person in the world coordinate system *X_w_Y_w_Z_w_* is measured by reading a smart navigation system with a built-in high frequency (HF) band RFID tag. The RFID reader is attached to the ankle of the visually impaired person. A smart navigation system is installed in the passage for the visually impaired person. The visually impaired person hangs a smartphone around their neck. The smartphone is equipped with a monocular camera in addition to the acceleration sensor, angular velocity sensor, and geomagnetic sensor to measure the posture of the smartphone. A monocular camera image, the position of the visually impaired person, and the posture of the smartphone are uploaded to a server on the cloud via 5G. The server analyzes the uploaded image and finds the coordinates (*x*^(*n*)^*_si_*, *y*^(*n*)^*_si_*, *z*^(*n*)^*_si_*) of the pedestrians in the camera coordinate system *X_s_Y_s_Z_s_*. The gaits of the pedestrians are obtained from the coordinates of the pedestrians’ skeletons, the position of the visually impaired person, and the posture of the smartphone. The proposed system learns the gaits of pedestrians and identifies objects by machine learning. The objects are pedestrians walking in the same direction as the visually impaired person, oncoming pedestrians, and steps. The identified object is mapped to geographic information. The type and location of the object and the safe route are downloaded from the server to the visually impaired person’s smartphone via 5G. The visually impaired person walks along the recommended passages.

To protect the privacy of visually impaired people, this system collects only personal information that is sufficient for the service. We obtain consent to collect personal information when installing the proposed system on a smartphone. In addition, the privacy of pedestrians is protected by discarding the video shot with the camera after detecting objects and recommending a safe route.

### 3.2. Sensor Selection

In positioning, the difference between indoor and outdoor is the number of shields. In a room, radio waves are absorbed/reflected/transmitted depending on the material such as a ceiling, pillar, and wall. Since there is no shielding in the sky outside, the system can capture radio waves from satellites. WIFI and beacons are generally used for indoor positioning methods. GPS is often used outdoors. RFID is expensive, but it is the most accurate compared to other positioning methods. Therefore, in this research, the position of visually impaired people was measured using RFID. In obstacle detection using a camera, the difference between indoors and outdoors is the change in illuminance and infrared rays due to sunlight. RGB-D cameras can have significantly reduced accuracy outdoors. In addition, if the illuminance changes rapidly in a short time, the obstacle detection accuracy by the camera will be significantly reduced. The OpenPose [27] used in this research can acquire a human skeleton using a monocular camera even outdoors. The skeleton can be estimated even if a part of the pedestrian’s body is hidden by a shield. A monocular camera is mounted on a general smartphone. Visually disabled people can use this system at low cost simply by installing this application on their smartphones. Therefore, it can be expected to spread widely to visually impaired people. 

### 3.3. Positioning of Visually Impaired People Using RFID

A visually impaired person can grasp their current position in the world coordinate system by wearing an RFID reader on the ankle and walking on the smart navigation system [4]. Smart navigation consists of tiles and HF band RFID tags. RFID tags are spread under the tiles. RFID tags are assigned unique identifications (IDs). When the RFID reader is brought closer to the RFID tag in the smart navigation system, the unique ID assigned to the RFID tag is read. By associating this unique ID with the coordinates of the world coordinate system, the current position in the world coordinate system is grasped. The resolution of smart navigation system is determined by the size of the passive RFID tag. In this research, a 5 cm RFID tag was built into a smart navigation system. Therefore, the resolution of the smart navigation system was 5 cm. 

### 3.4. Extraction of Pedestrian Skeleton from Monocular Camera Images

**Figure 3:**
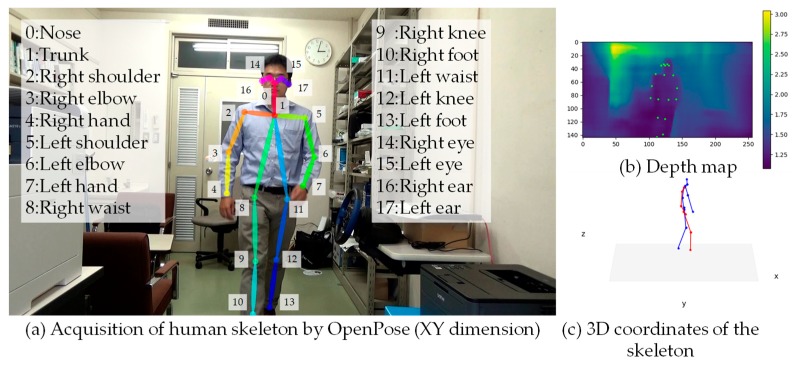
Joint parts, depth map, and skeleton are acquired with OpenPose.

A pedestrian’s skeleton in the camera coordinate system is acquired by capturing a front view using a smartphone monocular camera hanging on the neck of a visually impaired person. The coordinates of the pedestrian’s skeleton (*x*^(*n*)^*_s_*, *y*^(*n*)^*_s_*, *z*^(*n*)^*_s_*) in the camera coordinate system are extracted using OpenPose. OpenPose calculates the position of the skeletons of a total of 18 pedestrian trunks, legs, arms, and heads from monocular camera images (Figure 3). 

The coordinates of the pedestrian’s skeleton in the camera coordinate system are converted into the coordinates of the pedestrian’s skeleton in the world coordinate system from the position of the visually impaired person and the posture of the smartphone as follows:

(1) $$L_{Wi}^{\left(n\right)}\left(t\right)=M_{s}\left(t\right)L_{si}^{\left(n\right)}\left(t\right)$$

(2) $$M_{s}\left(t\right)=\left(\begin{array}{cc} R\left(\varphi ,\theta ,\psi \right) & \begin{array}{c} x\\ y\\ z \end{array}\\ \begin{array}{ccc} 0 & 0 & 0 \end{array} & 1 \end{array}\right)$$

where *L^(n)^_wi_ (t)* = [*x*^(*n*)^*_wi_ y*^(*n*)^*_wi_ z*^(*n*)^*_wi_*]*^T^* is the coordinate of the skeleton *i* of the *n*th pedestrian in the world coordinate system at a certain time *t*, *L^(n)^_si_(t)* = [*x*^(*n*)^*_si_*, *y*^(*n*)^*_si_*, *z*^(*n*)^*_si_*]*^T^* is the coordinate of the skeleton *i* of the *n*th pedestrian in the camera coordinate system at a certain time *t*, *M_S_*(*t*) is the homogeneous coordinate transformation matrix, *R*(*φ,θ,ψ*) is the *XYZ* rotation matrix, and *x*, *y*, *z* are the translational components. The *XYZ* rotation matrix is calculated from the posture of the smartphone. *x*, *y*, and *z* are calculated from the position of the smartphone. The posture of the smartphone is calculated from the values of the acceleration sensor, angular velocity sensor, and geomagnetic sensor built into the smartphone. The location of the smartphone is the same as the location of the visually impaired person. Therefore, *x* and *z* are the coordinates of the visually impaired person in the world coordinate system obtained by smart navigation. *y* is the distance from the ground to the smartphone wearing part.

### 3.5. Object Identification Based on Human Flow

The passage is divided into rectangular areas. The types of objects in each area are identified by machine learning. The types of objects are people walking in the same direction as visually impaired people, oncoming pedestrians, and steps. The objective variable for machine learning is the type of the object. The system calculates the relative coordinates of the trunk and each part, the coordinates of the trunk’s *Y_w_*-axis, and the speed of the trunk’s *X_w_*-axis and *Z_w_*-axis of the pedestrian’s skeleton. The explanatory variables are the mean, standard deviation, and maximum and minimum of these values. The machine learning is Random Forest. Random Forest outputs the result by the voting results of *N* decision trees. Random Forest outputs variable importance. This variable importance indicates a variable that is effective for identification. Therefore, Random Forest is a useful tool for analysis.

### 3.6. Object Identification Based on Human Flow

**Figure 4:**
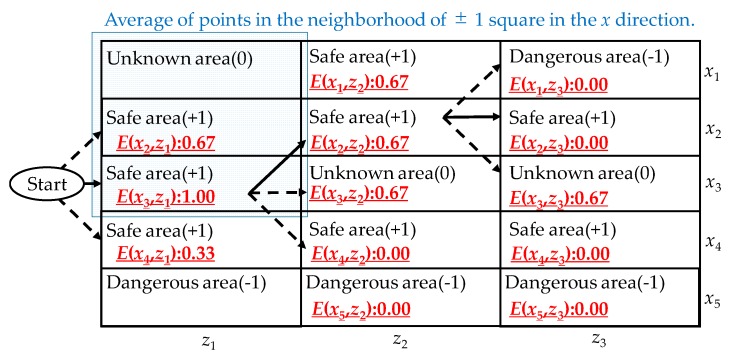
Recommending a safe route by a brute-force search based on the average score of the target area and surrounding areas.

As shown in Figure 4, the safe route is determined by a brute-force search. 

The areas assign points based on the identified results in Section 3.5 as follows:

(3) $$\delta \left(x,z\right)=\{\begin{array}{c} 1\;if\;Safe\;area\\ -1\;if\;Dangerous\;area\\ 0\;if\;Unknown\;area \end{array}$$

A safe area is an area where pedestrians are walking in the same direction as visually impaired people. A dangerous area is an area identified as containing oncoming pedestrians or steps. An unknown area is an area where the pedestrian’s skeleton cannot be obtained. The system sums the scores of the area of ± 1square in the *x_i_* direction of the area to be evaluated. The sum *E*(*x_j_,z_k_*) is a score of the area to be evaluated.

(4) $$E\left(x_{j},z_{k}\right)=\overset{1}{\underset{m=-1}{\sum }}\delta \left(x_{i+m},z_{k}\right)$$

A route that maximizes *E*(*x_j_,z_k_*) is recommended. The recommended route satisfies the following conditions:

1. Dangerous areas are not recommended;
2. Unknown areas are not recommended;
3. If a straight route and a turning route have the same score, a straight route is recommended.

If the routes available to the visually impaired person are only unknown areas, switch to the existing object identification method. A safe route is a passage where pedestrians can walk. Therefore, “under construction” areas can be avoided at the stage of recommending a safe route in addition to steps and oncoming pedestrians.

## 4. Experiment Results

### 4.1. Environment and Evaluation

**Figure 5:**
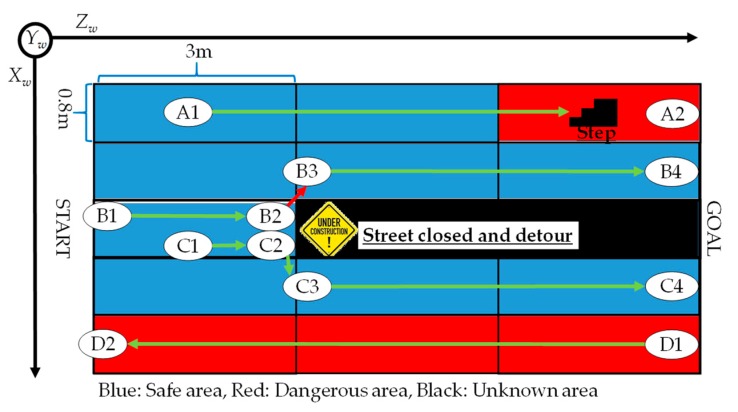
Experimental environment for evaluating the accuracy of identification and recommendation.

In the experiment, we verified the accuracy of object identification and the recommendation of a safe route based on human flow. To the best of the authors’ knowledge, the object detection and safe route recommendation using human flow in this research has not been studied so far. Therefore, the purpose of this experiment was to detect the object when the human flow was stably measured, and to verify the accuracy when searching for the optimum route using the object detection result. However, human detection methods and route-search algorithms are not the main proposals in this research. Therefore, an existing method was used as a human detection method and a route-search algorithm. The human skeleton was detected using OpenPose. OpenPose can estimate the position of a skeleton hidden by an object from a human skeleton model even when an occlusion problem occurs. A brute-force search was used for the route-search algorithm. When a brute-force search has an optimal solution in the solution space, the optimal solution can be obtained. The brute-force method is more computationally intensive than a metaheuristic algorithm such as ant colony optimization and genetic algorithms. The route-search problem belongs to NP-hard. However, the route set in this experiment had a small number of combinations, and real-time performance was not impaired even if the brute-force search was used. Therefore, the brute-force method was adopted in this research because an optimal solution was required. Figure 5 shows the experimental environment. 

In Figure 5, blue is a safe area, red is a dangerous area, and black is an unknown area. The experimental environment was 4 m in the *X_w_*-axis and 9 m in the *Z_w_*-axis. The steps were 0.6 m in the *X_w_*-axis, 0.4 m in the *Z_w_*-axis, and 0.6 m in the *Y_w_*-axis. The height of the steps was 0.3 m per step. As an “under construction” area, obstacles of 1 m in the *X_w_*-axis, 0.6 m in the *Z_w_*-axis, and 1 m in the *Y_w_*-axis were installed. The lighting used was general white lighting. In addition, the experimental environment was controlled so that outside light did not enter. The safety route update frequency was 100 msec. In the experiment, four pedestrians, A, B, C, and D, walked on different routes. Pedestrian A went straight, went up the steps, and then went down the steps (A1–A2). Pedestrian B avoided the “under construction” area to the left and went straight (B1–B4). Pedestrian C avoided the “under construction” area to the right and walked near pedestrian D (C1–C4). Pedestrian D was an oncoming pedestrian. Pedestrian D went straight on route D1–D2. The experimental environment of this research was adjusted so that the occlusion problem occurred. For example, the “under construction” areas were hidden by pedestrian B and pedestrian C at the start. Pedestrian D was hidden by pedestrian C. The stairs were hidden by pedestrian A and pedestrian B. The visually impaired people hung their smartphones from their necks. The visually impaired people then walked along the recommended route. The sensor values and images of the smartphones were acquired. The experiment was conducted 24 times. A smart navigation system was installed in the experimental environment. In this experiment, an RFID reader (AS14000USB manufactured by Artfinex) was used. The frequency of RFID was in the 13.56 Hz HF band. The communication distance of RFID was 3.0 cm to 5.0 cm. The smartphone operating system was Android. The frame rate of the smartphone camera was 10 fps. The camera resolution was 1280 × 720 pixels. Since the purpose of this research was to verify the accuracy of identification and route recommendation, a notebook personal computer (PC) was directly connected to a smartphone for processing. The central processing unit (CPU) of the notebook PC was i7-9750H (six cores, 2.60 GHz), the memory was 32 GB, and the graphics board was RTX2070. A 10-fold cross-validation was used to evaluate the object identification accuracy. Accuracy was used as an evaluation value as follows:

$$Accuracy=\frac{Number\;of\;correctly\;identified\;for\;each\;class}{Sample\;size\;for\;each\;class}$$

### 4.2. Results

Table 1 shows the identification results of each object. 

The columns in Table 1 represent observation results and the rows represent identification results. From Table 1, the oncoming pedestrians were completely identified. The pedestrians walking in the same direction as the visually impaired people were identified with an accuracy of 0.93. The pedestrians walking in the same direction as the visually impaired people were misidentified as oncoming pedestrians and steps. The steps were identified with an accuracy of 0.82. The steps were misidentified as pedestrians walking in the same direction as visually impaired people. The average accuracy of identification was 0.92. Table 2 shows the top 10 important variables for identification. Variables with high mean decrease Gini in Table 2 are important for identification.

The variable importance was high in the order of the trunk speed in the *Z_w_*-axis and *X_w_*-axis, and the trunk coordination in the *Y_w_*-axis. Table 2 shows that the speed and coordinates of the trunk are important variables. Table 3 shows the results of nonparametric multiple comparison tests using the Steel-Dwass method for the variables in Table 2. The test was performed assuming that there was a difference between the two groups. We obtained statistical knowledge by testing with the Steel-Dwass method. The significance level of the Steel-Dwass method is *p*-value = 0.01. The column in Table 3 represents the test results between the two groups. Items with significant differences are shown in bold in Table 3.

Since pedestrians walking in the same direction as visually impaired people and oncoming pedestrians have different walking directions, there was a significant difference in the speed of the trunk in the *Z_w_*-axis in distinguishing between pedestrians walking in the same direction as a visually impaired person and an oncoming pedestrian. There was a significant difference in the standard deviation of the trunk speed in the *X_w_*-axis. The test results represent the change in speed in the *X_w_* direction when pedestrian B and pedestrian C turned to the “under construction” areas. Since the walking speed for going up and down steps is different from normal, pedestrians walking in the same direction as visually impaired people and steps had a significant difference in the maximum trunk speed and standard deviation in the *Z_w_*-axis. There was a significant difference in the coordinates of the trunk in the *Y_w_*-axis since the coordinates of the trunk in the *Y_w_*-axis moved while going up the steps. Oncoming pedestrians and steps had significant differences in trunk speed in the *Z_w_*-axis, the standard deviation of trunk speed in the *X_w_*-axis, and trunk position in the *Y_w_*-axis. This occurred for the same reason as the difference between the same pedestrians and steps. This was the same reason as the difference between the pedestrians walking in the same direction as visually impaired people and steps.

**Figure 6:**
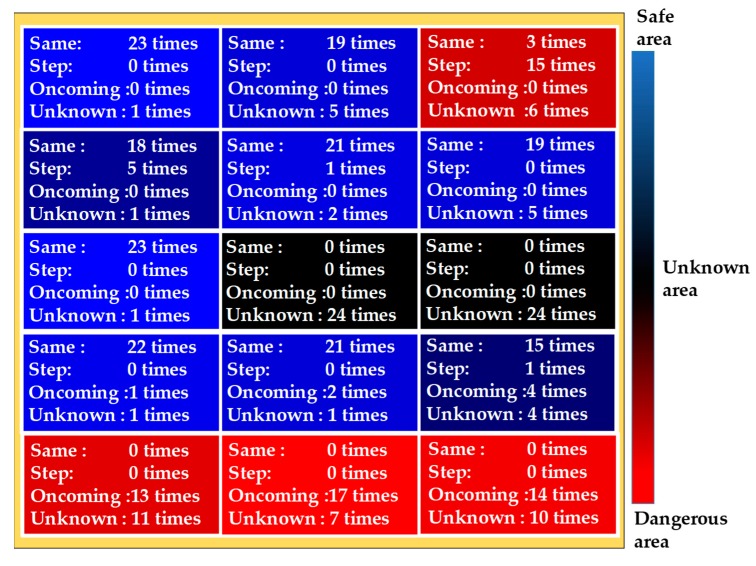
Identification results by area.

Figure 6 shows the identification results for each area. 

From Figure 5, Table 1, and Figure 6, the area (*x*_1_, *z*_3_) was misidentified as a pedestrian moving in the same direction as a visually impaired person and as steps. This was caused by vibration noise on the Y-axis during walking. The case where a pedestrian moving in the same direction as a visually impaired person was misidentified as an oncoming pedestrian was near the area of the oncoming pedestrian. Therefore, misidentification was caused by positioning accuracy.

**Figure 7:**
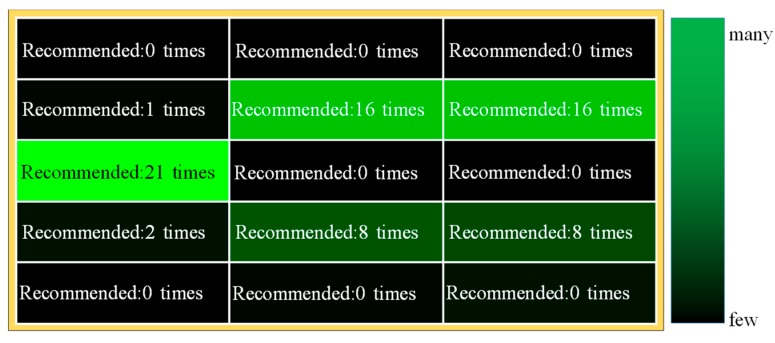
Identification results by area.

Figure 7 shows the recommended routes based on the identification results. 

In the identification results, there were cases where safe areas were misidentified as dangerous areas, but the areas on the recommended route were all safe areas. Furthermore, all recommended routes avoided “under construction” areas. The recommended route was the route that pedestrian B and pedestrian C walked. In this experiment, the recommended method areas were not all unknown areas, so the existing method did not change. Pedestrian B’s route was recommended more than pedestrian C’s route since the area around pedestrian C was a dangerous area. Therefore, the proposed system is effective. 

### 4.3. Contribution and Limitation

In this experiment, we verified the object detection accuracy by human flow in an environment where the illuminance does not change. Moreover, the recommendation accuracy of the safe route was verified by a brute-force search that obtained the optimal solution for the route search. In this experiment, the most likely situation was created in the laboratory. These verification results show that visually impaired people can be guided by finding the optimum safe route from the human flow in an indoor environment where the illuminance does not change. It was a great contribution of this research to show that it is possible to recommend a safe route using human flow in an indoor environment. As a related research that is closest to the approach of this research, Gupta et al. [28] propose the concept of a system that selects a leader from the crowd and navigates according to that leader. The related research report on the accuracy of reader selection and human detection. However, since the related research did not actually navigate visually impaired people, the effect of navigation by human flow was not quantitatively verified. Leung et al. [29] estimate the surface with SLAM in a dynamically changing environment. In this related research, assuming small camera variations, the system use the v-disparity domain to find ground support points for least squares plane fitting. All these approaches require at least a portion of the ground to be visible for each estimation step. Strong camera movements are not considered. As long as the camera pose change between frames is small enough and the ground is still the dominant plane, the tracking plane parameter will succeed. In related research, steps were not detected. This paper clarified these questions through experiments. In this paper, a leader was not detected, and a safe route was obtained from the route of an unspecified number of pedestrians. Therefore, compared to related research, this research is novel and effective.

On the other hand, from Figure 6, this research requires attention as the detection accuracy of human skeletons affects object detection and safe route recommendation. Therefore, when the proposed system is used outdoors where the illuminance changes, the detection accuracy of the human skeleton is reduced, so that the accuracy of object detection and safe route recommendation is likely to be reduced. There are various cases of routes such as intersections and pedestrian crossings. It is necessary to consider these factors in the future for practical application. 

## 5. Conclusions and Future Works

In this research, we constructed the experimental environment by setting up “under construction” areas, steps, oncoming pedestrians, and pedestrians walking in the same direction as visually impaired people and verified the accuracy of object identification and safe route recommendation using human flow in an environment where pedestrians can be detected stably. As a result, steps hidden by pedestrians could be identified from the pedestrian’s gait and route with an average accuracy of 0.92. In addition, “under construction” areas could be avoided 100%. It is useful that this research showed that a 100% recommendation accuracy can be achieved if the optimal solution for the safe route is obtained using human flow. It was shown that the coordinates of the trunk in the direction of a pedestrian and the trunk in the *Y_w_*-axis are effective in identifying them. Furthermore, as a result of scoring and recommending areas, the percentage of recommended routes that included dangerous areas was 0%. 

In the future, since there are various objects and walking patterns in the real world, we will increase the patterns of the experimental environment. As a countermeasure against vibration noise during walking, a wheeled cane will be developed and a device that does not generate vibration during walking will be developed. In addition, we will communicate with the server on the cloud under 5G and verify whether it can be processed in real time as well. If another pedestrian is hidden by a pedestrian, the human detection accuracy will be reduced. The detection accuracy of pedestrians changes with changes in illumination. We will examine the effects of the decrease in human detection accuracy on the object detection accuracy and safety route recommendation accuracy of the proposed system. Various methods other than OpenPose have been proposed for detecting skeletons. We think that it is better to select a method that is robust against illuminance changes and robust against the occlusion problem. In addition, 5G is highly straight forward and is currently operated in a limited environment. IEEE802.11ax (WIFI6) is a high-speed indoor communication network. We will consider the use of these high-speed communication networks. In addition, there are areas where communication is possible only in the environment of low-speed communication networks such as 4G/ Long Term Evolution (LTE). For low-speed communication networks, it is necessary to maintain the real-time property by reducing the frame rate or lowering the resolution. We will investigate these switching methods in the future. In this method, positioning was performed using RFID. However, if a positioning with the same accuracy as RFID that can be implemented easily at low cost is developed in the future, it can be replaced. In this research, it was not necessary to calculate so many routes. Therefore, we sought the best route with brute force. When finding a more complicated route, it is difficult to implement in real time if it is with brute force, so it is necessary to find a suboptimal solution using a metaheuristic method. We would like to examine route-search methods in accordance with the situation.

## Figures and Tables

**Table 1:** Identification results of the pedestrians walking in the same direction as the visually impaired people, and oncoming pedestrians and steps.

		Observation Results			Accuracy
		Same	Oncoming	Step	
Identification results	Same	181	0	3	0.93
	Oncoming	7	45	0	1.00
	Step	7	0	15	0.83
Average					0.92

**Table 2:** Top 10 of the important variables for identification.

Variable	Mean Decrease Gini
Maximum speed in the z-axis of trunk	50.9
Average speed in the z-axis of trunk	47.9
Minimum speed in the z-axis of trunk	32.2
Standard deviation speed in the z-axis of trunk	15.7
Standard deviation speed in the x-axis of trunk	13.3
Minimum speed in the x-axis of trunk	12.5
Maximum speed in the x-axis of trunk	10
Average of y-axis trunk coordinate	5.46
Average of x-axis eye relative coordinate	5.13

**Table 3:** Results of multiple comparison tests on the variables in Table 2.

Variable	*p*-Value		
	Same vs. Oncoming	Same vs. Step	Oncoming vs. Step
Maximum speed in the z-axis of trunk	**0.00**	**0.00**	**0.00**
Average speed in the z-axis of trunk	**0.00**	0.02	**0.00**
Minimum speed in the z-axis of trunk	**0.00**	0.54	**0.00**
Standard deviation speed in the z-axis of trunk	**0.00**	**0.00**	**0.00**
Standard deviation speed in the x-axis of trunk	**0.00**	0.33	**0.00**
Minimum speed in the x-axis of trunk	**0.00**	0.73	**0.00**
Maximum speed in the x-axis of trunk	0.20	**0.01**	**0.00**
Average of y-axis trunk coordinate	0.94	**0.00**	**0.01**
Average of x-axis eye relative coordinate	0.09	0.05	0.02

## References

[1] World Heath Organization Visual Impairment and Blindness. http://www.who.int/mediacentre/factsheets/fs282/en/.

[2] Dunai L.D., Lengua I.L., Tortajada I., Simon F.B. Obstacle detectors for visually impaired people. Proceedings of the 2014 IEEE International Conference on Optimization of Electrical and Electronic Equipment (OPTIM).

[3] Fernandes H., Costa P., Filipe V., Paredes H., Barroso J. (2019). A review of assistive spatial orientation and navigation technologies for the visually impaired. Univ. Access Inf. Soc..

[4] Alghamdi S., van Schyndel R., Khalil I. (2014). Accurate positioning using long range active RFID technology to assist visually impaired people. J. Netw. Comput. Appl..

[5] Asano M., Kajiwara Y., Shimakawa H. (2015). Supporting for Visually Handicapped to Walk Around with RFID Technologies. Sens. Transducers.

[6] Durrant-Whyte H., Bailey T. (2006). Simultaneous localization and mapping: Part I. IEEE Robot. Autom. Mag..

[7] Yeboah M.O., Kuada E., Sitti M., Govindan K., Hagan H., Miriam M.C. Design of a Voice Guided Ultrasonic Spectacle and Waist Belt with GPS for the Visually Impaired. Proceedings of the 2018 IEEE 7th International Conference on Adaptive Science & Technology (ICAST).

[8] Nair V., Tsangouri C., Xiao B., Olmschenk G., Seiple W.H., Zhu Z. (2018). A Hybrid Indoor Positioning System for Blind and Visually Impaired Using Bluetooth and Google Tango. J. Technol. Pers. Disabil..

[9] Großwindhager B., Rath M., Kulmer J., Bakr M.S., Boano C.A., Witrisal K., Römer K. (2018). SALMA: UWB-based Single-Anchor Localization System using Multipath Assistance. Proceedings of the 16th ACM Conference on Embedded Networked Sensor Systems.

[10] Fallah N., Apostolopoulos I., Bekris K., Folmer E. (2013). Indoor human navigation systems: A survey. Interact. Comput..

[11] Zhang X., Yao X., Zhu Y., Hu F. (2019). An ARCore Based User Centric Assistive Navigation System for Visually Impaired People. Appl. Sci..

[12] Yang Z., Duarte M.F., Ganz A. A Novel Crowd-Resilient Visual Localization Algorithm Via Robust Pca Background Extraction. Proceedings of the 2018 IEEE International Conference on Acoustics, Speech and Signal Processing (ICASSP).

[13] Mahmud N., Saha R.K., Zafar R.B., Bhuian M.B.H., Sarwar S.S. Vibration and voice operated navigation system for visually impaired person. Proceedings of the 2014 IEEE International Conference on Informatics, Electronics & Vision (ICIEV).

[14] Sadi M.S., Mahmud S., Kamal M.M., Bayazid A.I. Automated walk-in assistant for the blinds. Proceedings of the 2014 IEEE International Conference on Electrical Engineering and Information & Communication Technology.

[15] Blasch B., Corbett M., Penrod W. (2005). A Master Trainer Class for Professionals in Teaching the UltraCane Electronic Travel Device. J. Vis. Impair. Blind..

[16] Ye C., Hong S., Qian X., Wu W. (2016). Co-robotic cane: A new robotic navigation aid for the visually impaired. IEEE Syst. Man Cybern. Mag..

[17] Yang K., Wang K., Cheng R., Hu W., Huang X., Bai J. (2017). Detecting traversable area and water hazards for the visually impaired with a pRGB-D sensor. Sensors.

[18] Parikh N., Shah I., Vahora S. Android smartphone based visual object recognition for visually impaired using deep learning. Proceedings of the 2018 IEEE International Conference on Communication and Signal Processing (ICCSP).

[19] Yusuke Kajiwara K.O. (2016). High-Speed Detection of Down Staircase for Visually Impaired People Using Illuminance of Point Pattern. Sens. Mater..

[20] Schwarze T., Lauer M., Schwaab M., Romanovas M., Bohm S., Jurgensohn T. An intuitive mobility aid for visually impaired people based on stereo vision. Proceedings of the IEEE International Conference on Computer Vision Workshops.

[21] Chillaron M., Dunai L., Fajarnes G.P., Lengua I.L. Face detection and recognition application for Android. Proceedings of the IECON 2015-41st Annual Conference of the IEEE Industrial Electronics Society.

[22] Jacobson R.D. (1998). Cognitive mapping without sight: Four preliminary studies of spatial learning. J. Environ. Psychol..

[23] Jacquet C., Bellik Y., Bourda Y. (2006). Electronic locomotion aids for the blind: Towards more assistive systems. Intelligent Paradigms for Assistive and Preventive Healthcare.

[24] Kajimoto H., Kanno Y., Tachi S. Forehead electro-tactile display for vision substitution. Proceedings of the EuroHaptics.

[25] González-Mora J.L., Rodriguez-Hernaindez A.F., Burunat E., Martin F., Castellano M.A. Seeing the world by hearing: Virtual Acoustic Space (VAS) a new space perception system for blind people. Proceedings of the 2006 2nd IEEE International Conference on Information & Communication Technologies.

[26] Mohebbi R., Gray R., Tan H.Z. (2009). Driver reaction time to tactile and auditory rear-end collision warnings while talking on a cell phone. Hum. Factors.

[27] Cao Z., Simon T., Wei S.E., Sheikh Y. Realtime multi-person 2d pose estimation using part affinity fields. Proceedings of the IEEE Conference on Computer Vision and Pattern Recognition.

[28] Gupta A., Khandelwal S., Gandhi T. Blind navigation using ambient crowd analysis. Proceedings of the 2018 IEEE 8th International Advance Computing Conference (IACC).

[29] Leung T.S., Medioni G. Visual navigation aid for the blind in dynamic environments. Proceedings of the IEEE Conference on Computer Vision and Pattern Recognition Workshops.

